# Potential Global Distribution of the Invasive Mosquito *Aedes koreicus* under a Changing Climate

**DOI:** 10.3390/tropicalmed8100471

**Published:** 2023-10-10

**Authors:** Qing Liu, Jing-Wen Xie, Ming Wang, Yu-Tong Du, Zi-Ge Yin, Ning-Xin Zhou, Tong-Yan Zhao, En-Jiong Huang, Heng-Duan Zhang

**Affiliations:** 1 State Key Laboratory of Pathogen and Biosecurity, Beijing Institute of Microbiology and Epidemiology, Beijing 100071, China; 15290116829@163.com (Q.L.); xiejw990222@163.com (J.-W.X.); wming0108@163.com (M.W.); du2009yutong@163.com (Y.-T.D.); 15810335316@163.com (Z.-G.Y.); 15755498580@163.com (N.-X.Z.); tongyanzhao@126.com (T.-Y.Z.); 2 The School of Public Health, Fujian Medical University, Fuzhou 350000, China; 3 Fuzhou International Travel Health Care Center, Fuzhou 350001, China

**Keywords:** invasive mosquito, *Aedes koreicus*, MaxEnt, potential distribution

## Abstract

Invasive alien species are a growing threat to natural systems, the economy, and human health. Active surveillance and responses that readily suppress newly established colonies are effective actions to mitigate the noxious consequences of biological invasions. *Aedes (Hulecoeteomyia) koreicus* (Edwards), a mosquito species native to East Asia, has spread to parts of Europe and Central Asia since 2008. In the last decade, *Ae. koreicus* has been shown to be a competent vector for chikungunya virus and *Dirofilaria immitis*. However, information about the current and potential distribution of *Ae. koreicus* is limited. Therefore, to understand the changes in their global distribution and to contribute to the monitoring and control of *Ae. koreicus*, in this study, the MaxEnt model was used to predict and analyze the current suitable distribution area of *Ae. koreicus* in the world to provide effective information.

## 1. Introduction

*Aedes (Hulecoeteomyia) koreicus* (Edwards), a new invasive mosquito species in Europe, appears to have a daytime habit of feeding on human blood [1,2] and is a vector of many diseases. Experimental transmission of Japanese encephalitis virus (JEV) by *Ae. koreicus* was reported as early as 1967 [3]. It has been experimentally proven that *Ae. koreicus* is most likely a competent vector of dog *Dirofilaria immitis* and is of potential relevance in the natural cycle of the parasite [4]. In addition, some studies showed that experimentally infected *Ae. koreicus* specimens were able to transmit Zika virus and chikungunya virus under laboratory conditions [5,6]; the mosquitoes were capable of transmitting CHIKV at a higher temperature (27 °C ± 5 °C), with no transmission at 24 °C ± 5 °C; and the infection rate at the higher temperature (68.2%) was four times higher than that at the lower temperature (17.6%) [5]. Regarding the Zika virus, the vector competence was relatively low (4.7%) and temperature dependent; in particular, ZIKV transmission occurred only at a higher temperature [5,6].

Some studies have shown that at the end of the annual season, when the day becomes shorter, *Ae. koreicus* females are able to lay desiccated and cold-resistant eggs that can survive during the winter and hatch in the spring similar to other females of *Aedes* species, and *Ae. koreicus* can survive in cold and dry environments, which allows this species to avoid competition with other mosquito species and has considerable advantages in breeding and transmission [7,8]. Most studies suggest that *Ae. koreicus* originated in North Korea, China, Japan, and Russia [8,9,10], and since 2008, when the species was first reported in Belgium [11], it has been found in several European countries. The first discovery of *Ae. koreicus* in Italy was in 2011 [12]. Then, in mid-2015, a mosquito specimen collected in southern Germany was identified as *Ae. koreicus* [13]. In 2016, the first occurrence reports of *Ae. koreicus* were published in Hungary [14], the first reported *Ae. koreicus* in Slovenia was in 2013 [15], and the first reported *Ae. koreicus* in Austria and Kazakhstan was in 2018 [16,17]. In 2021, the first record of *Ae. koreicus* in the Czech Republic and the Netherlands was reported [18,19]. In the above countries, *Ae. koreicus* has established stable populations except in Switzerland, Slovenia, and the Netherlands. Several studies indicate that *Ae. koreicus* is well adapted to urban settlements, where a variety of artificial containers can be found as breeding sites in gardens and urban areas, and similar to other species of the genus *Aedes*, the female *Ae. koreicus* lays eggs in artificial containers, so the introduction of *Ae. koreicus* could be due to the trade of small containers, ornamental plants, or used tires and has spread throughout Europe through international trade [8,20,21]. *Ae. koreicus* has now invaded Europe from Asia and settled in areas partially occupied by other mosquito species after *Ae. albopictus*, *Aedes aegypti*, and *Ae. japonicus* [8].

Although there is very little published evidence on its vector status, *Ae. koreicus* is recognized as a potential vector of arboviruses due to its known role as a vector of CHIKV, ZIKV, and *D. immitis* under laboratory conditions. According to the WHO, a total of 89 countries and territories have reported evidence of mosquito-transmitted Zika virus infection, with the first local mosquito-transmitted ZIKV disease cases reported in Europe in 2019 and ZIKV outbreak activity detected in India in 2021 (https://www.who.int/news-room/fact-sheets/detail/zika-virus, accessed on 29 September 2023). Chikungunya was first identified in the United Republic of Tanzania in 1952 and subsequently in other countries, such as Africa and Asia, and has now been identified in over 110 countries in Asia, Africa, Europe, and the Americas (https://www.who.int/news-room/fact-sheets/detail/chikungunya, accessed on 29 September 2023). Meanwhile, the global expansion of exotic mosquito species has intensified with the development of international trade and increased movement of people; coupled with climate warming and urbanization, this trend will further increase in the future [22,23,24,25]. The invasion of *Ae. koromikos* not only has an impact on the biodiversity of the invaded areas but also poses a potential risk to local public health safety. For this reason, the possible involvement of a new mosquito species as a vector is crucial to set up the necessary preventive measures to contain the spread of infections. Understanding its distribution is important for the formulation of an *Ae. koreicus* control strategy in invaded areas.

Species distribution models (SDMs) are models that correlate information on species distribution samples with information on corresponding environmental variables to derive the relationship between species distribution and environmental variables and apply this relationship to the area under study to estimate the distribution of target species [26]. MaxEnt is a commonly used species distribution model.

The maximum entropy model (MaxEnt) is based on the maximum entropy theory, which treats species and their growing environment as a system, determines the stable relationship between species and the environment by calculating the state parameters when the system has maximum entropy, and estimates the distribution of species [27]. Several scholars [28,29,30] have demonstrated that the MaxEnt model is a successful tool for analyzing the potential distribution areas of species, and some have also used species distribution models to predict the potential distribution areas of mosquitoes.

The prediction of the potential habitat of *Ae. aegypti* used MaxEnt ecological niche modeling in Tanzania [31], showing that the potential distribution of *Ae. Aegypti* will shift toward the central and northeastern parts with intensification in areas around all major lakes in the future. The MaxEnt model was also applied to predict the potential global distribution of *Ae. albopictus* and *Ae. aegypti* by Kamal [32] et al., showing that under the 2050 and 2070 climate scenarios, *Ae. albopictus* would expand in the northern United States, parts of southern Canada, and eastern Europe, and *Ae. aegypti* would expand to southeastern Australia. The predicted potential distribution of *Ae. albopictus* in China by Liu et al. [33] using the MaxEnt model showed that the potential distribution area of *Ae. albopictus* in China will expand westward due to future climate change under the SSP126 climate scenario. The distribution potential of the two species was different. The above examples show that the MaxEnt model can predict the potential distribution of species under variable climatic conditions by combining species occurrence data with natural environmental data and that its results are theoretically more accurate and reliable.

Current research on the monitoring and distribution of *Ae. koreicus* mosquitoes mainly focuses on a particular country or region, and there are very few studies on predicting the global distribution of *Ae. koreicus* mosquitoes, showing a gap in the understanding of changes in their global distribution. In addition, *Ae. koreicus* is recognized as a potential vector of arboviruses due to its proven role as a vector of the viruses CHIKV and ZIKV, which are important threats to humans. As the species continues to spread, new threats to public health will emerge, and analyzing the likelihood of further incursions by *Ae. koreicus* is therefore particularly important. In this study, we chose to use the MaxEnt model to predict and analyze the current suitable global distribution area of *Ae. koreicus* to provide effective information for *Ae. koreicus* monitoring and control.

## 2. Materials and Methods

### 2.1. Mosquito Occurrence Data

**Figure 1:**
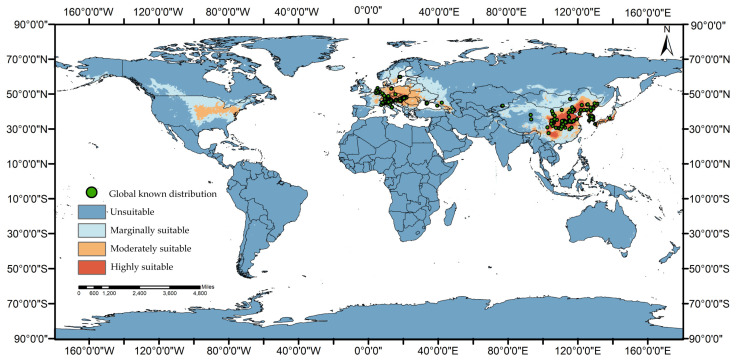
Global predicted suitable areas under the current climatic conditions for *Ae. koreicus*.

The *Ae. koreicus* occurrence data were collected from two sources. First, a comprehensive and systematic literature retrieval was performed. We used the keyword “*Ae. koreicus*” searched in PubMed and CNKI (China National Knowledge Infrastructure) (Supplementary Table S1). The second source was the Global Biodiversity Information Facility (GBIF) database (https://www.gbif.org/occurrence/search?taxon_key=1652150&occurrence_status=present, accessed on 26 August 2023), which contains global occurrence records of many species, and we used “*Ae. koreicus*” as the keyword to include only species names “*Ae. koreicus*” or “*Hulecoeteomyia koreica*” distribution points and eliminated duplicates with those found in PubMed and CNKI. A total of 410 distribution records were integrated and entered into the corresponding Excel sheet in the model input file format (Supplementary Table S2), with column 1 set to the species name, column 2 set to the longitude of the species distribution, and column 3 set to the latitude of the species distribution (both longitude and latitude were written in decimal format), and saved in a csv format for later use. The sampling location can be seen in Figure 1.

### 2.2. Climate Data

The 19 climate factors, topographic elevation data, and climate data for 2021–2040 and 2041–2060 under the SSP (shared socioeconomic pathway) 126 climate scenarios used in the model projections were downloaded from WorldClim (https://www.worldclim.org/, accessed on 26 August 2023), with both current and future variables having a high resolution of 2.5 arcmin (approx. 5 km × 5 km). The 19 climate factors included the annual mean temperature, maximum temperature of the warmest month, minimum temperature of the coldest month, and annual precipitation (Table 1). These data were closely related to the distribution of species and were necessary for prediction. The environmental data were converted by GIS software to ASCII data recognizable by the model.

The species distribution points and environment were imported into maxent.jar software and run once to obtain the contribution rate of each environmental factor, to reduce the autocorrelation between variables and avoid overfitting the model predictions. The species distribution and climate data were imported into ArcMap 10.4.1 software, and the climate information of the *Ae. koreicus* distribution points was extracted using the “sampling” function and imported into SPSS 24.0 software for correlation analysis. Among the two environmental factors, the one with a higher contribution in the MaxEnt modeling process was selected from the two environmental factors with a correlation coefficient *r* > 0.9 [34]. Finally, we selected Bio01, Bio02, Bio03, Bio05, Bio06, Bio07, Bio08, Bio12, Bio13, Bio14, Bio15, Bio19, and elevation as environmental variables for the *Ae. koreicus* distribution model.

### 2.3. Ecological Niche Model

Maximum entropy theory originates from statistical mechanics and is a general method for making predictions or inferences from incomplete information. The idea behind the MaxEnt model is to estimate the target probability distribution by finding the probability distribution of maximum entropy under certain constraints. The model software used for modeling in this paper was MaxEnt 3.4.1.

### 2.4. Model Construction

#### 2.4.1. Model Construction under Current Climate Conditions

(1) Import species distribution data and environmental variable data into maxent.jar software, and set the following parameters: the proportion of training data is 75%, the output format is selected as ASC raster format, the number of model repetitions is set to 1, and other parameters are set to the software defaults.

(2) Open the GIS software, import the results of model in ArcMap10.4.1 to overlay the prediction map and define the projection, and open the Spatial Analyst tool inside the Arc toolbox to perform the reclassification process. The classification method is the natural breaks (Jenks) method, and the distribution area is divided into four levels: unsuitable region, lowly suitable region, moderately suitable region and highly suitable region. Finally, the map is created.

#### 2.4.2. Model Construction under the SSP1-2.6 Climate Scenario

(1) Import the species distribution data and current and future environmental variable data into maxent.jar software and set the parameters: the proportion of training data is 75%, the output format is selected as ASC raster format, the number of model repetition runs is set to 1, and other parameters are set to the software default.

(2) Open the GIS software, input the results of the model in ArcMap10.4.1 and define the projection, and open the Spatial Analyst tool inside the Arc toolbox to perform the reclassification process. The classification method is the natural breaks (Jenks) method, and the distribution area is divided into four levels: unsuitable, marginally suitable, moderately suitable, and highly suitable. Finally, the map is created.

#### 2.4.3. Model Evaluation

To test the predictive ability of MaxEnt in this study, the receiver operating characteristic (ROC) curve was used. The AUC (area under the ROC curve) value is the area enclosed by the ROC curve and the horizontal coordinate; it indicates the accuracy of the model, and the value ranges from 0 to 1. A more accurate model is indicated by a higher AUC value.

## 3. Results

### 3.1. Global Predicted Suitable Areas under the Current Climatic Conditions for Ae. koreicus

According to the prediction results, the global area of *Ae. koreicus* was 5,453,100 km^2^, of which 7,146,900 km^2^ was highly suitable (Table 2), mainly in China, Japan, North Korea, South Korea, and some areas of Europe (Figure 1). The low-suitability area covered 3,529,200 km^2^ and was primarily located in the United States of America in North America, France, Germany, Poland, the Czech Republic, and Ukraine in Europe and China, Russia, South Korea, North Korea, and Japan in Asia. The lowly suitable area was 7,890,800 km^2^ and was mainly found in Canada and the United States in North America, Norway, Sweden, and Finland in Europe and China, Mongolia, Russia, and Japan in Asia, and some parts of Laos, India, and Pakistan were also included in the lowly suitable area.

### 3.2. Model Evaluation

**Figure 2:**
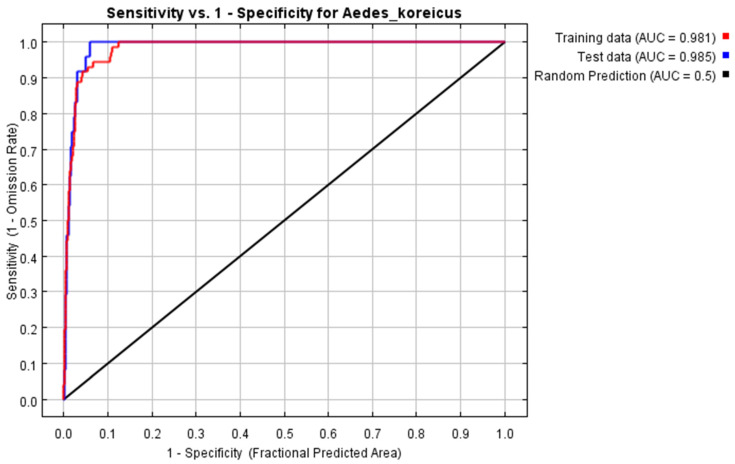
The receiver operating characteristic (ROC) curve.

The AUC value of the MaxEnt model was 0.985 (Figure 2), which showed that the prediction accuracy was good, indicating that the present study based on the MaxEnt model to predict the potential distribution of *Ae. koreicus* in the world has high credibility.

### 3.3. Global Predicted Suitable Areas the SSP1-2.6 Climate Scenario for Ae. koreicus

**Figure 3:**
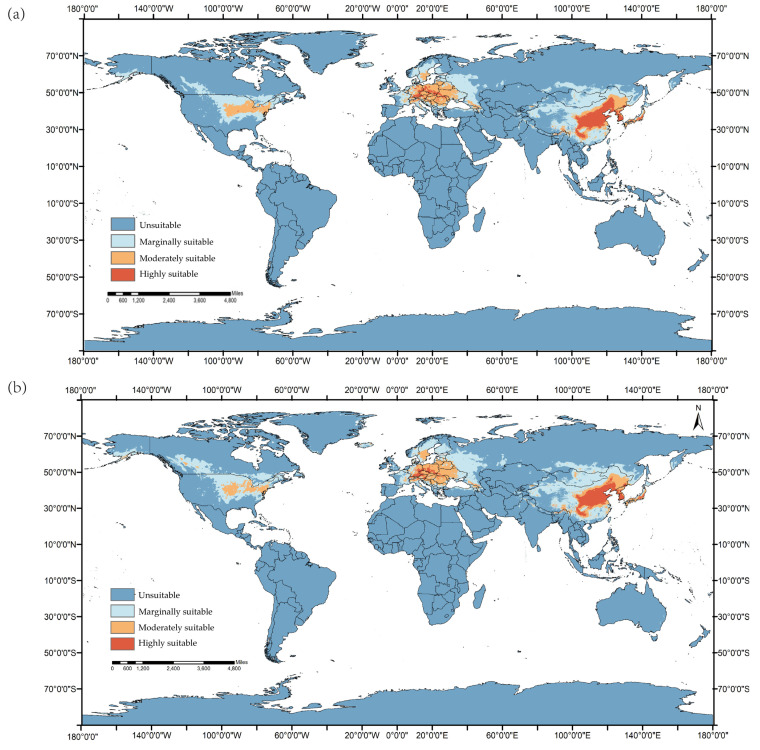
Global predicted suitable areas the SSP1-2.6 climate scenario for *Ae. koreicus*: (**a**) 2021–2040 and (**b**) 2041–2060.

According to the predictions of the MaxEnt model (Figure 3, Table 3), the total global area suitable for *Ae. koreicus* will increase by 3,434,200 km^2^ in 2021–2040 under the SSP1-2.6 climate scenario compared with the current prediction, and the global total suitable area for *Ae. koreicus* will increase by 5,069,800 km^2^ in 2041–2060 compared with the current prediction. 

### 3.4. Filtering for Environmental Factors

**Figure 4:**
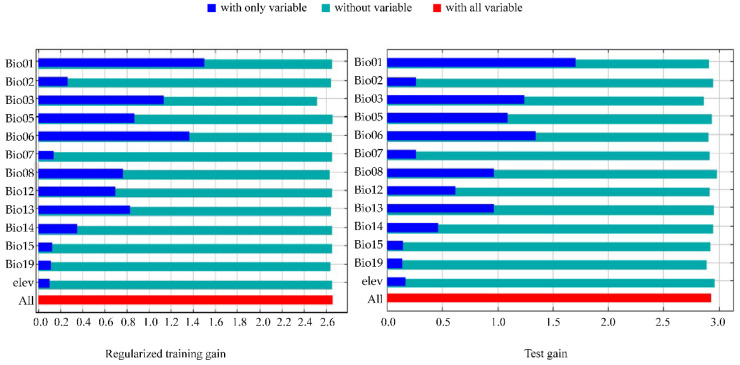
Jackknife test of environmental variable importance for the MaxEnt model.

**Figure 5:**
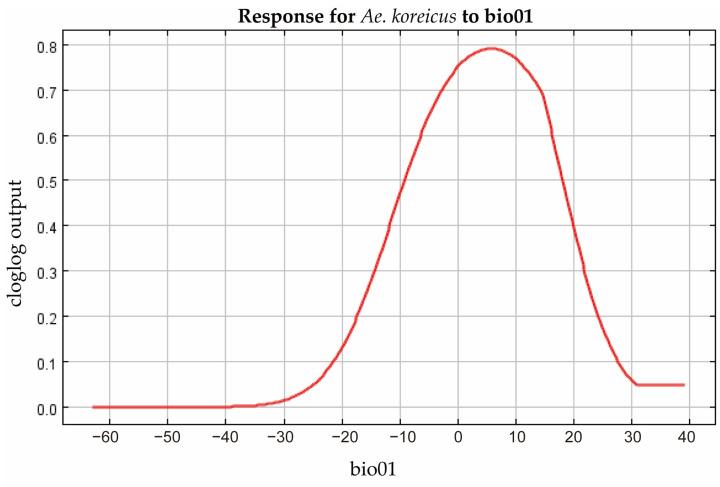
Response curve of Bio1 in the potential distribution model of *Ae. koreicus*.

**Figure 6:**
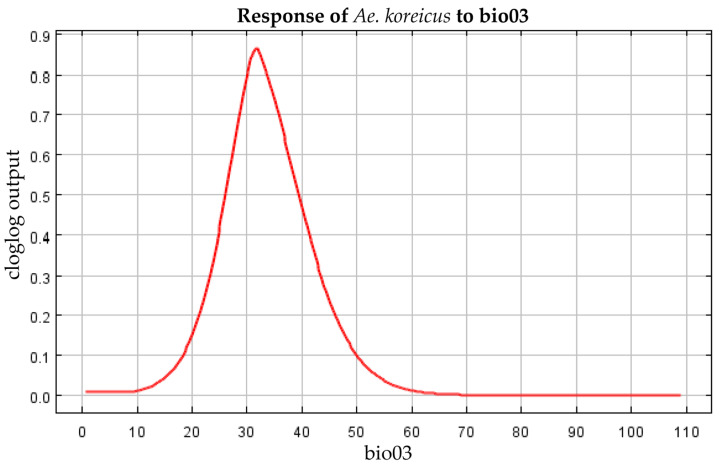
Response curve of Bio3 in the potential distribution model of *Ae. koreicus*.

**Figure 7:**
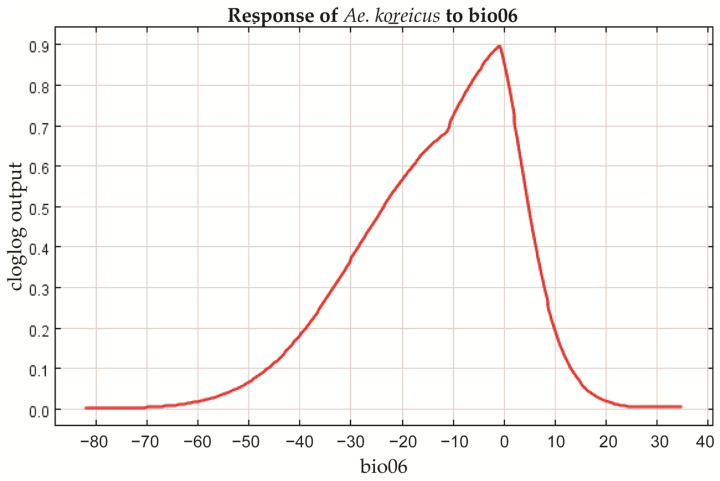
Response curve of Bio6 in the potential distribution model of *Ae. koreicus*.

The contribution of each environmental factor to the MaxEnt modeling is shown in Table 4, in which Bio01 (mean annual temperature), Bio13 (precipitation in the wettest month), Bio03 (isothermality), and Bio06 (minimum temperature in the coldest month) have made a major contribution to the spread of *Ae. koreicus*. The analysis of the contribution values of the environmental variables to the MaxEnt model was also performed by the jackknife method, and the results of both the regularized training gain and the test gain indicated that the most important environmental factor was Bio1, followed by Bio6 and Bio3 (Figure 4). The response curves of the three environmental factors are shown in Figure 5, Figure 6 and Figure 7. The Bio1 response curve indicates that the probability of the presence of *Ae. koreicus* in North Korea was highest when the mean annual air temperature was approximately 5 °C. The Bio3 response curve indicates that the probability of the presence of *Ae. koreicus* in North Korea showed a trend of increase followed by a decrease with increasing isothermality. The Bio3 response curve indicates that the probability of the presence of *Ae. koreicus* in North Korea showed an increasing trend followed by a decreasing trend with increasing isothermality, and the probability of the presence of the mosquito was highest when the isothermality was approximately 32. The Bio6 response curve shows that the probability of distribution of *Ae. koreicus* in North Korea showed a trend of increasing and then decreasing, and the highest probability was found when the minimum temperature of the coldest month increased to 0 °C and then began to decrease.

## 4. Discussion

The predicted results of this study showed that under the SSP126 climate scenario, the highly suitable area of *Ae. koreicus* in the European region showed a tendency to expand toward the north in the future. These characteristics suggest *Ae. koreicus* might continue to expand across the continent. A few months after the first discovery of the species in Italy in 2011, until 2012, *Ae. koreicus* was found in 37 municipalities (39.4%) and was detected in 40.2% of places and in 37.3% of larval habitats monitored, in a range of altitudes from 173 to 1250 miles above sea level in northeastern Italy [35]. The results clearly showed that *Ae. koreicus* is well established in northeastern Italy and confirmed the invasive potential of this mosquito. Then in the summer of 2013, for the first time, *Ae. koreicus* was found in the Swiss Italian border region of Ticino–Lombardy and southern European Russia [36,37]. Two years later, a mosquito specimen collected in mid2015 in southern Germany was identified as *Ae. koreicus*. Subsequently, the first occurrence reports of *Ae. koreicus* were published in Hungary (2016) [14], Austria (2018) [38], and the Netherlands (2022) [19]. The distribution area of *Ae. koreicus* is indeed widening, under the influence of global warming and heavier international trade and travel in Europe. 

The most important environmental factor for the distribution of *Ae. koreicus* was Bio1, followed by Bio6 and Bio3, all three of which are temperature variables. Mosquitoes are small-bodied poikilotherms, meaning that the ambient temperature is the main abiotic factor limiting their development, reproduction, ecological niche, and, therefore, geographical distribution [24,39]. Experimental evidence suggests that *Ae. koreicus* can survive at temperatures ranging from 10 °C to 28 °C [40]. The prediction results also showed that the highly suitable areas for *Ae. koreicus* are mainly in northern Italy and southern Germany near the Alps in Europe, the Yangtze and Yellow River basins in China, and the area surrounding Mount Fuji in Japan. In 2011, *Ae. koreicus* was found in a small village of Belluno Province in northern Italy, and this is the first report in Italy of the introduction of the exotic mosquito *Ae. koreicus*. Then, in 2015, the first record of *Ae. koreicus* in southern Germany was reported. These areas for the establishment of *Ae. koreicus* were characterized by mid-high annual average temperatures, between 10 °C and 15 °C [40]. *Ae. koreicus* can overwinter in the form of diapause eggs and can better adapt to urban environments [41,42,43]. The temperature was reported to play a major role in determining the habitat suitability for *Ae. koreicus* [41]. These results are consistent with the trends in the response curves of temperature-related environmental factors derived in this study, which increased and then decreased. In addition, *Ae. koreicus* is able to lay diapause eggs that survive the winter and hatch in the spring, allowing adult *Ae. koreicus* to persist from late summer into autumn [38,44]. For these reasons, it can be better adapted to lower temperatures, which facilitates its invasive colonization of hilly and alpine foothills [8]. Although the biology and ecology of *Ae. koreicus* are poorly known, the results of our study on the potential distribution of *Ae. koreicus* may suggest that the distribution of this species seems to be more dependent on temperature changes.

“Shared socioeconomic pathways” (SSPs) are a group of new emissions scenarios driven by different socioeconomic assumptions. Many of the new SSP scenarios were used for CMIP6, providing scientists with a wider choice to model the potential future. SSP1-2.6, SSP2-4.5, SSP3-7.0, and SSP5-8.5 are different scenarios showing the annual carbon dioxide emissions up to 2100 under this scenario [45]. Under the SSP1-2.6 climate scenario, the global mean annual land precipitation is predicted to change in the range of 0.0–6.6 percent, and the global mean surface air temperature (GSAT) is predicted to increase in the range of 0.5 °C–1.5 °C in the period 2081–2100 compared with 1995–2014 [46]. Some scholars have proposed [47] that the global average surface temperature (GMST) is increasing at a rate of 0.2 ± 0.1 °C per decade. The GMST is estimated to be 1.5 °C higher in the 2030–2050 period than it was during the pre-industrial period (the reference period is 1850–1900). Warmer seasons, which are more likely to occur in the future because of climate change, might extend the breeding time and therefore increase the abundance of *Ae. koreicus* in the world. As we can see in the prediction results under the SSP1-2.6 scenario, the global total suitable area for *Ae. koreicus* will increase in 2021–2040 and 2041–2060, and the highly suitable area will remain distributed in northern Italy and southern Germany near the Alps in Europe, the Yangtze and Yellow River basins in China, and the area around Mount Fuji in Japan.

Although no case of *Ae. koreicus*-transmitted disease has been reported, *Ae. koreicus* is recognized as a potential vector of arboviruses due to its proven role as a vector of CHIKV, ZIKV, and *D. immitis*. Moreover, the cold-tolerant biology of *Ae. koreicus* gives it an advantage in terms of spreading and establishing its population. Between 2007 and 2012, many autochthonous cases of dengue and chikungunya were reported in Europe [48]. CHIKV was first isolated in the 1950s from patients in Tanzania, and then the virus began to spread widely over the last decade [49]. In 2013, approximately 2.4 million cases of dengue fever were reported in the Caribbean and Central and South America, and 773 travel-associated cases were reported in the United States of America, with 48 cases of local transmission reported in the continental United States [50]. Some scholars have suggested that there is great potential for CHIKV to spread quickly in North America, much like West Nile virus did more than a decade ago [49,51]. The results of our predictions show that northeastern North America is a marginally and moderately suitable area for *Ae. koreicus*, which highlights the potential risks of invasion of *Ae. koreicus* into North America. As a defined vector of CHIKV, it is impossible to completely ignore the role it may play in the future spread of the virus. Furthermore, the predicted results in this study showed that given the risks associated with the presence of *Ae. koreicus*, we cannot ignore the need to monitor and control this species.

In addition, the *Ae. koreicus* population has been found to be resistant to etofenprox and permethrin with possible resistance to deltamethrin but was susceptible to chlorpyrifos in a recent study [52]. A study on microbiological analyzes on *Ae. koreicus* in the field discovered that both *Wolbachia* and *Asaia* bacteria were detected in *Ae. koreicus* collected in northeastern Italy, as *Asaia* and *Wolbachia* are considered new tools for symbiotic control, which, given the increasing development of resistance in *Aedes* mosquitoes, also supports their use for innovative control strategies against invasive *Ae. koreicus* [53]. 

The rapid spread of *Ae. koreicus* in some places (e.g., Italy) may have originated from one or a few initial infections, suggesting that its further spread may be under control if certain control measures are taken. Research on changes in the distribution of *Ae. koreicus* is lacking, and studying changes in its distribution can provide effective information for its monitoring and control. Based on the results of this study, it is possible to reduce the risk of spread and public health risks by implementing effective control measures in areas where *Ae. koreicus* populations are already established and by initiating regular surveillance in risk areas where *Ae. koreicus* may spread.

## 5. Conclusions

This study provides, to our knowledge, the first experimental data on the global distribution of the invasive mosquito *Ae. koreicus*. According to this study, the areas suitable for *Ae. koreicus* survival are currently distributed mainly in in China, Japan, North Korea, South Korea, and some areas of Europe, and most of the highly suitable areas are distributed in China. Northeastern North America is the marginally and moderately suitable area for *Ae. koreicus*. Under the SSP1-2.6 climate scenario, the total global suitable area for *Ae. koreicus* will increase in the next forty years. Therefore, the species might further spread in the absence of control measures. For this reason, investigations on the main modes and routes of dispersal of this invasive species and an increase in monitoring efforts should be prioritized.

## Figures and Tables

**Table 1:** Relevant environmental variables used for modeling.

Bioclimate	Description
Bio01	Annual mean temperature
Bio02	Mean diurnal range (mean of monthly (max temp–min temp))
Bio03	Isothermality (Bio2/Bio7) (×100)
Bio04	Temperature seasonality (standard deviation ×100)
Bio05	Max temperature of warmest month
Bio06	Min temperature of coldest month
Bio07	Temperature annual range (Bio5–Bio6)
Bio08	Mean temperature of wettest quarter
Bio09	Mean temperature of driest quarter
Bio10	Mean temperature of warmest quarter
Bio11	Mean temperature of coldest quarter
Bio12	Annual precipitation
Bio13	Precipitation of wettest month
Bio14	Precipitation of driest month
Bio15	Precipitation seasonality (coefficient of variation)
Bio16	Precipitation of wettest quarter
Bio17	Precipitation of driest Quarter
Bio18	Precipitation of warmest quarter
Bio19	Precipitation of coldest quarter
Elev	Elevation

**Table 2:** Global predicted suitable areas under the current climatic conditions for *Ae. koreicus*.

Level	10^4^ km	%
Unsuitable	13,615.60	91.38
Marginally suitable	789.08	5.30
Moderately suitable	352.92	2.37
Highly suitable	142.40	0.96

**Table 3:** Global predicted suitable areas the SSP1-2.6 climate scenario for *Ae. koreicus*.

Level	2021–2040 (10^4^ km^2^)	2041–2060 (10^4^ km^2^)
Unsuitable	133,369.00	13,108.62
Lowly suitable	1002.71	1233.59
Moderately suitable	373.39	394.24
Highly suitable	154.91	163.55

**Table 4:** Percent contribution of the environmental variables on the distribution in the MaxEnt model.

Environmental Variables	Percent Contribution
Bio01	24.3
Bio13	23.9
Bio03	14.8
Bio06	13.5
Bio19	7.5
Bio08	7.1
Bio14	5.9
Bio02	2.1
Bio15	0.5
Elev	0.2
Bio05	0.1
Bio12	0
Bio07	0

## Data Availability

Not applicable.

## Supplementary Materials

The following supporting information can be downloaded at https://www.mdpi.com/article/10.3390/tropicalmed8100471/s1: Table S1: Retrieved literature; Table S2: Geographical coordinates of selected distribution points.
